# First-Principles Surface Stress Calculations and Multiscale Deformation Analysis of a Self-Assembled Monolayer Adsorbed on a Micro-Cantilever

**DOI:** 10.3390/s140407435

**Published:** 2014-04-23

**Authors:** Yu-Ching Shih, Chuin-Shan Chen, Kuang-Chong Wu

**Affiliations:** 1 Institute of Applied Mechanics, National Taiwan University, Taipei 10617, Taiwan; E-Mails: ycstone@ntu.edu.tw (Y.-C.S.); wukc@mail.iam.ntu.edu.tw (K-C.W.); 2 Department of Civil Engineering, National Taiwan University, Taipei 10617, Taiwan

**Keywords:** multiscale modeling, density functional theory, finite element method, micro-cantilever sensors

## Abstract

Micro-cantilever sensors are widely used to detect biomolecules, chemical gases, and ionic species. However, the theoretical descriptions and predictive modeling of these devices are not well developed, and lag behind advances in fabrication and applications. In this paper, we present a novel multiscale simulation framework for nanomechanical sensors. This framework, combining density functional theory (DFT) calculations and finite element method (FEM) analysis, is capable of analyzing molecular adsorption-induced deformation and stress fields in the sensors from the molecular scale to the device scale. Adsorption of alkanethiolate self-assembled monolayer (SAM) on the Au(111) surface of the micro-cantilever sensor is studied in detail to demonstrate the applicability of this framework. DFT calculations are employed to investigate the molecular adsorption-induced surface stress upon the gold surface. The 3D shell elements with initial stresses obtained from the DFT calculations serve as SAM domains in the adsorption layer, while FEM is employed to analyze the deformation and stress of the sensor devices. We find that the micro-cantilever tip deflection has a linear relationship with the coverage of the SAM domains. With full coverage, the tip deflection decreases as the molecular chain length increases. The multiscale simulation framework provides a quantitative analysis of the displacement and stress fields, and can be used to predict the response of nanomechanical sensors subjected to complex molecular adsorption.

## 1. Introduction

Nanomechanical sensors have attracted considerable interest, as they are a promising tool for real-time and label-free detection of chemical gases and biomolecules [1–7]. These molecular adsorbates introduce surface stresses upon the detective surface layer and sequentially produce measurable displacement and stress fields in the sensors [8,9]. For cantilever-shaped nanomechanical sensors, the output signals are often measured as tip deflections using a position-sensitive photodetector [6] or as strain/stress changes near clamping regions using a Wheatstone bridge [6,10].

The sensitivity of the induced surface stress dominates the performance of cantilever-shaped nanomechanical sensors. Understanding the physical mechanisms of adsorption-induced surface stress and their influences on the overall displacement and stress fields are the key to designing next-generation nanomechanical sensors. Surface stresses due to molecular adsorption often arise from two main sources: weak inter-adsorbate interactions and strong adsorbate–substrate interactions [1,9,11]. Inter-adsorbate interactions between electrically neutral adsorbates are usually attractive, as described by van der Waals or London dispersion forces, and will induce tensile surface stresses on the surface layers of nanomechanical sensors. On the other hand, inter-adsorbate interactions between charged adsorbates are usually repulsive, as described by electrostatic forces, and will induce compressive surface stresses on the surface layers of nanomechanical sensors. The strong adsorbate-substrate interactions occur because of chemical bond formation or chemical reaction. This interaction often leads to surface charge redistribution. A substrate gaining electrons from the adsorbates often results in tensile surface stresses, while transferring charges to the adsorbates often gives rise to compressive surface stresses. The overall adsorption-induced surface stresses are the net effect of the aforementioned mechanisms.

Quantitative analysis of the displacement and stress fields of a nanomechanical sensor due to the adsorption-induced surface stress remains a theoretical challenge [9]. The complexity of the competing mechanisms requires detailed molecular modeling. On the other hand, device-level calculations of the displacement and stress fields are beyond the reach of molecular modeling, but can be satisfactorily described by continuum mechanics. For example, analytical formulas for the displacement of the cantilever subjected to surface stress have been derived [12–14]. It thus naturally calls for multiscale modeling to couple the molecular model with the continuum description. Dareing and Thundat developed a semi-analytical model for calculating adsorption-induced surface stresses based on atomic interactions [15]. To simplify the derivation, the model was restricted to the case of a single atomic layer adsorption with a simple Lennard-Jones potential. Chen *et al.* [16–18] derived a multiscale method to couple a continuum description with first-principles density functional theory (DFT) calculations. This method linked atomic contributions with kinematic constraints imposed by continuum mechanics and provided a pathway to study detailed physics of adsorption-induced surface stresses. However, one drawback of this multiscale method is that the deformation field of the devices needs to be known *a priori*. The aim of this paper is to generalize the method by removing the kinematic constraints imposed on the device deformation field.

In this study, we propose a multiscale simulation framework for nanomechanical cantilever sensors based on DFT calculations and finite element method (FEM) analysis. DFT calculations are used to compute the induced surface stress of molecular adsorption on the molecular recognition layer. The calculated surface stresses are then used in the FEM analysis to resolve the deformation and stress fields of the nanomechanical sensors. A gold-coated cantilever sensor exposed to alkanethiolate self-assembled monolayers (SAM) is used to demonstrate the applicability of the proposed multiscale framework.

## 2. Multiscale Simulation Framework

**Figure 1.**
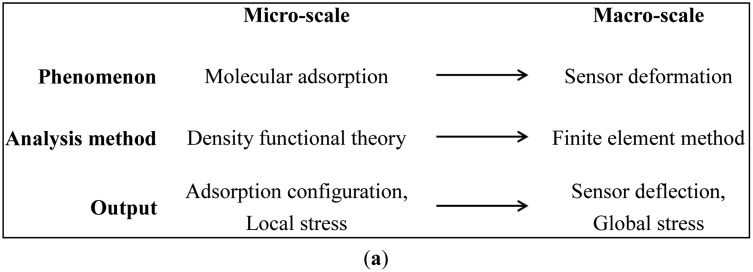

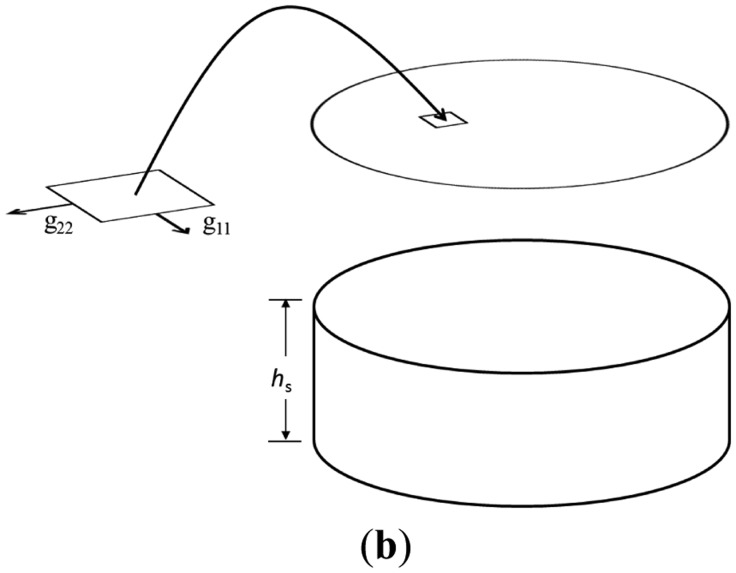
(**a**) Multiscale simulation framework: bridging the molecular simulation with adsorption-induced deformation of nanomechanical sensors; (**b**) The link between DFT and FEM. The DFT surface stresses (g_11_ and g_22_) are substituted as initial stresses in a section of film depicted as a 2D circle. The thickness *h_s_* of the substrate (illustrated as a cylinder but could be any shape) is much greater than that of the film. The thin film and substrate parts of the sensors are simulated together in the FEM analysis.

Figure 1a illustrates the respective phenomena, analysis methods, and calculated outputs at the micro- and macro-scales for the proposed multiscale modeling framework. At the micro-scale (*i.e.*, the molecular level), we are concerned with molecular adsorption phenomena and use DFT calculations to obtain detailed molecular adsorption configurations and local surface stresses, g_11_ and g_22_, as shown in Figure 1b. At the macro-scale (*i.e.*, the device level), we are interested in resolving the deformation and stress fields of the sensors. We use FEM analysis with thin shell/solid modeling techniques to obtain sensor deflections and overall stress and strain distributions in the sensors with initial stresses obtained from the DFT calculations. These modeling methodologies are described in detail below using a gold-coated micro-cantilever sensor exposed to alkanethiolate SAMs.

### 2.1. Theoretical Description

**Figure 2.**
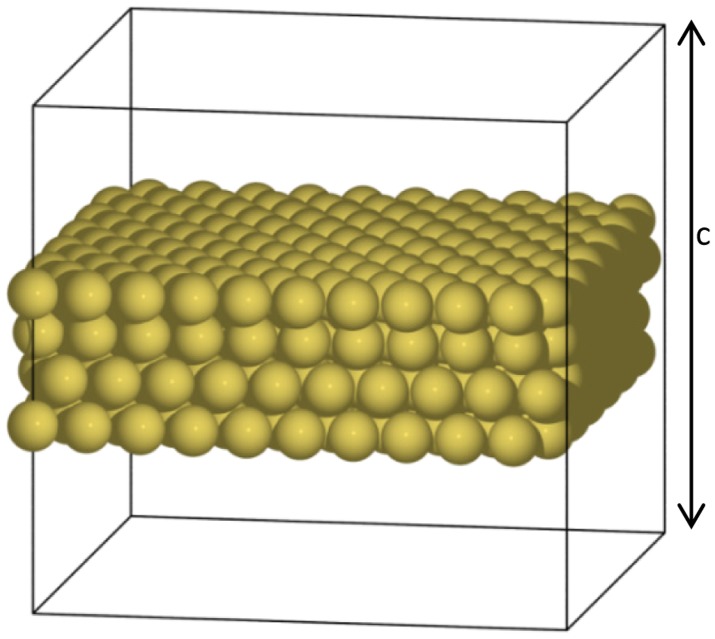
An illustration of the supercell model for a clean gold surface.

Firstly, we describe the theoretical background of this framework. The surface stress tensor is defined as:

(1) $$g_{\alpha \beta }=\frac{1}{A}\frac{\partial \left(\gamma A\right)}{\partial \varepsilon_{\alpha \beta }}$$

where *γ* is the surface energy per unit area, *A* is the surface area, and *ε_αβ_* denotes the strain tensor [19]. The indices *α* and *β* indicate directions in the surface plane; for example, indices of 11 and 22 are the principal stresses and *xx*, *yy*, and *xy* are the normal stresses and the shear stress, respectively. In DFT calculations, a supercell representative volume is used before and after molecular adsorption. For the clean gold surface shown in Figure 2, the surface energy per unit area can be written as:

(2) $$\gamma =\frac{1}{2A}\left[E_{s}-n\times E_{b}\right]$$

where *E_s_* refers to the total energy of the supercell, *E_b_* is the bulk energy per unit cell, and *n* indicates the number of gold atoms. The factor ½ accounts for the two equivalent surfaces in the supercell model.

The surface stress tensor for the clean gold surface can be obtained from Equations (1) and (2):

(3) $$g_{\alpha \beta }=\frac{\Omega }{A}\left(\sigma_{\alpha \beta }^{s}-n\sigma_{\alpha \beta }^{b}\right)$$

where $\sigma_{\alpha \beta }^{s}$ and $\sigma_{\alpha \beta }^{b}$ are the supercell and bulk stresses, respectively and Ω is the volume of the supercell. The forces and stresses in the representative volume can be obtained directly from DFT calculations using the Hellmann–Feynman theorem. If the bulk lattice constant is used appropriately, the bulk stress $\sigma_{\alpha \beta }^{b}$ is zero. Therefore, the surface stress can be further simplified to [20]:

(4) $$g_{\alpha \beta }=\frac{1}{2}c\sigma_{\alpha \beta }^{s}$$

where *c* is the height of the supercell along the surface normal. The formula can also be applied to a metal surface covered in molecules. The molecular adsorption-induced surface stress is then the difference in the stress between a clean and a molecule-covered surface.

According to several experimental observations, alkanethiolate molecules self-assemble into well-ordered, poly-crystalline monolayers on the Au(111) surface. For FEM analysis at the device level, we assume that each film element represents an alkanethiolate SAM domain on a gold surface, whose initial stress is equal to the induced surface stress of the representative volume obtained from DFT calculations. The nodal loads of each film element are the work balance of the body forces {**F**} in volume *V*, the surface tractions {Φ} on surface *S*, the strain {ε_0_}, and the initial stress{*g_αβ_*}. The element load vector is:

(5) $$\left\{{\text{r}}_{e}\right\}=\int {\left[\text{N}\right]}^{T}\left\{\text{F}\right\}dV+\int {\left[\text{N}\right]}^{T}\left\{\Phi \right\}dS+\int {\left[\text{B}\right]}^{T}\left\{\text{E}\right\}\left\{\varepsilon_{0}\right\}dV-\int {\left[\text{B}\right]}^{T}\left\{g_{\alpha \beta }\right\}dV$$

where [**N**], [**B**], and {**E**} are the shape function matrix, the strain-displacement matrix, and the material property matrix, respectively [21].

**Figure 3.**
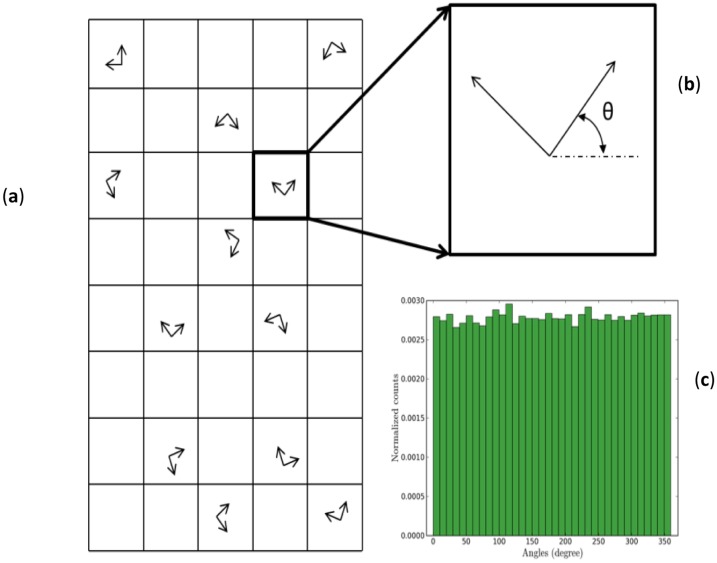
(**a**) An illustration of 30% coverage of randomly dispersed SAM domains; (**b**) An arbitrary angle θ is used to convert the principal stress into normal stress and shear stress components; (**c**) A normalized histogram plot of the uniformly distributed angles.

In general, the SAM orientations on the gold surface are complex and induced surface stresses from the SAM adsorption are anisotropic [22–24]. Here, we assume that SAM domains are randomly dispersed on the surface (as shown in Figure 3a) and oriented according to an arbitrary angle (as shown in Figure 3b). These arbitrary angles are distributed uniformly from 0 to 2*π* (as shown in Figure 3c) and are used to convert the principal stresses (*g*_11_ and *g*_22_) into normal stress components (*g*_xx_ and *g_yy_*) and shear stress component (*g_xy_*) in each shell element via Mohr's circle.

### 2.2. Density Functional Calculations

DFT calculations have been widely used to predict and estimate a great variety of material and molecular properties [25]. The calculations are *ab initio* since only atomic types and their spatial positions are required. All our DFT calculations were performed using the Vienna Ab initio Simulation Package (VASP) [26,27]. VASP uses a plane wave basis set to perform electronic structure calculations as well as quantum mechanical molecular dynamics from first principles. It has been widely used in solid-state physics and quantum chemistry fields.

**Figure 4.**
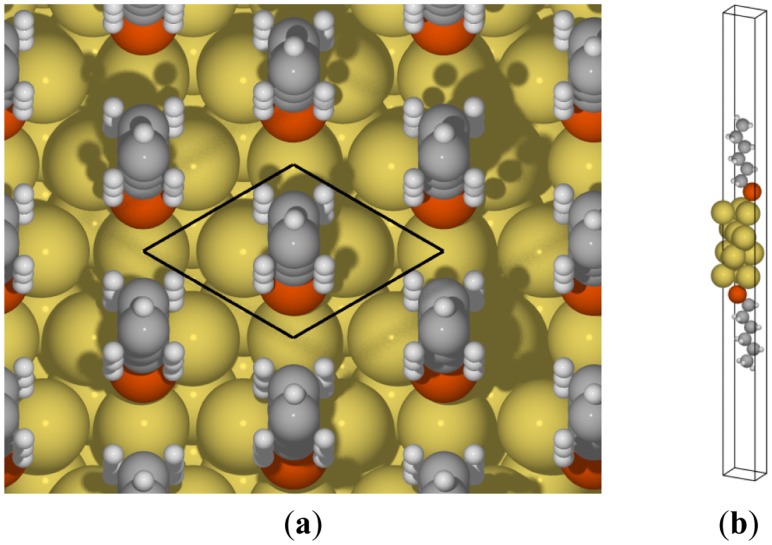
(**a**) Top view of the hexanethiolates on the Au(111) surface. Solid lines represent a $\left(\sqrt{3}\times \sqrt{3}\right)\text{R}30{}^{\circ}\text{Au}(111)$ surface unit cell. The gray, white, yellow, and orange spheres indicate carbon, hydrogen, gold, and sulfur atoms, respectively; (**b**) A 3D view of the DFT unit cell indicated in (a).

To obtain the induced surface stresses, we applied DFT calculations in two unit cells: An alkanethiolate-coated Au(111) surface and a clean Au(111) surface. For the clean gold surface, the unit cell consists of a $\left(\sqrt{3}\times \sqrt{3}\right)\text{R}30{}^{\circ}\text{Au}(111)$ surface slab of four Au atomic layers (each layer has three Au atoms) in the center with at least 16 Å vacuum layers on each side. For the molecule-coated surface, the same unit cell was used, and an alkanethiolate with a chain length of up to a hexanethiolate was added in the vacuum layer on both sides, as shown in Figure 4. Periodic boundary conditions were imposed on all the boundaries of the unit cells, to create infinite slabs in the surface plane and image slabs in the direction perpendicular to the slab surface. Therefore, it is important to use vacuum layers that are thick enough to force the electronic density to vanish at the boundaries above and below the slab so that two surfaces will not interact.

The projector augmented wave [28] and a 400 eV plane-wave cutoff were used. The Brillouin zone of the unit cells was meshed by a 16 × 16 × 1 Monkhorst–Pack *k*-points grid. To account for the effects of dispersion interactions on the adsorption of alkanethiolate to Au(111), which are major interaction forces between biomolecules, a recently developed fully non-local van der Waals density functional (vdW-DF) with an optB86b generalized gradient approximation (GGA) exchange functional was employed in the calculations. The vdW-DF exchange-correlation energy is of the form [29–32]:

(6) $$E_{xc}=E_{x}^{\text{GGA}}+E_{c}^{\text{LDA}}+E_{c}^{nl}$$

where $E_{x}^{\text{GGA}}$, $E_{c}^{\text{LDA}}$, and $E_{c}^{nl}$are the GGA exchange energy, the local-density approximation (LDA) correlation energy, and the non-local correction energy, respectively.

The unit cells, whose calculated equilibrium lattice constant is 4.14 Å, were kept fixed during atomic relaxation, and the optimized structures were obtained until the force on each atom was less than 0.01 eV/Å. After obtaining the optimized structures, we calculated the stress tensor of the slabs using the Hellmann–Feynman theorem [33–35]. Surface stresses were subsequently calculated using Equation (4). The adsorption-induced surface stress was obtained from the difference of the surface stresses of the two unit cells.

### 2.3. Finite Element Analysis

FEM analysis was employed in the device level simulation. A commercial finite element software, ABAQUS, with a thin shell/solid modeling technique was used to capture the deformation and stress fields of the sensors [36]. As shown in Figure 1b, we used a shell section and a solid section to represent the molecular adsorption layer and the rest of the sensor, respectively. A typical micro-cantilever biosensor, composed of gold and silicon nitride layers, with a length *L* of 206 μm and a width *W* of 60 μm was adopted. One end of the micro-cantilever was attached to a 20 μm × 100 μm support-block with the same material layers and additional silicon substrate.

**Figure 5.**
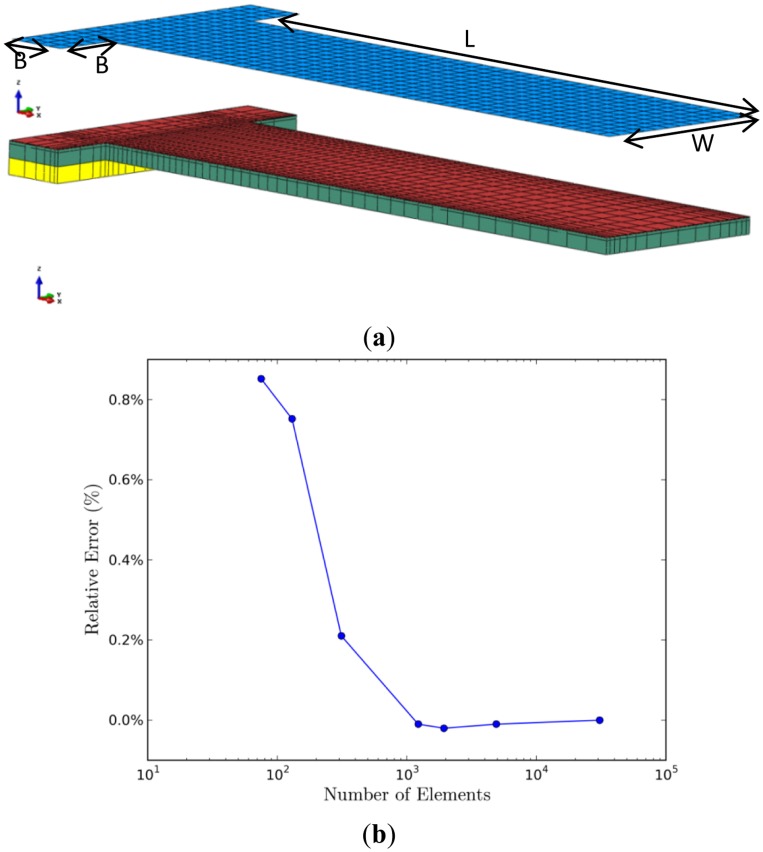
(**a**) Finite element models of the micro-cantilever. The upper part is the shell element section and the lower part is the solid element section. For clarity, the two parts are separated, the number of surface elements is reduced, and the direction of the surface normal is scaled for clarity. The colors blue, red, green, and yellow indicate the surface, gold, silicon nitride, and silicon sections, respectively. The beam has a length, *L*, of 206 μm and a width, *W*, of 60 μm. The support block has a length, *B*, of 20 μm and a width of 100 μm; (**b**) A mesh convergence study: the relative error *versus* the number of shell elements used in the model.

Figure 5a shows the finite element models of the cantilever sensor, which was modeled using 3D quadratic solid elements (C3D20R in ABAQUS), while the alkanethiolate SAM adsorption on Au(111) was modeled using 3D quadratic thin shell elements (S8R in ABAQUS). The surface section and solid section were modeled with 57,440 shell elements and 3,920 solid elements, respectively. The thickness of the shell element is the same as the distance between the center of the gold slab and the last carbon in the chain tail of the optimized alkanethiolate in the DFT unit cell, along the normal direction to the slab surface. The material properties and the thickness of each material layer are listed in Table 1.

We note that scanning tunneling microscope (STM) images suggest the adsorption layer of alkanethiol SAMs typically characterized by SAM domains with areas of close-packed molecules separated by domain boundaries [37]. The size of a shell element in the finite element model thus corresponds to the characteristic length of a SAM domain. It is then naturally to use a very small shell element to faithfully represent a SAM domain on the gold surface. The model shown in Figure 5a has 57,440 shell elements which corresponds to a 500 nm^2^ SAM domain. The number of shell elements is well beyond the needs of solution convergence for a micro-cantilever. For completeness, a convergence study was carried. Figure 5b plots the relative error *versus* the number of shell elements with the same coverage and distribution of SAM domains. Superior solution quality was found even for the mesh with much less shell elements.

The surface stresses obtained from the DFT calculations were applied as initial stresses in the shell elements. A surface-to-surface tie constraint was used to attach the shell section to the top surface of the solid section, so that two sections could be built separately but simulated together. Another advantage of this modeling technique is that it is not necessary to match the number of elements of the solid part with that of the shell section. This not only reduces computing time considerably, but also allows us to study the effects of SAM coverage and distribution on the deformation and stress distribution of the sensors. The support block's bottom and top faces along the surface normal were kept fixed, except for the face connected to the beam. The nonlinear geometry procedure in ABAQUS was applied in the static stress/strain analysis. Applying nonlinear geometry procedure in ABAQUS was meant for completeness but not mandatory since the typical deflections of a micro-cantilever are very small and the geometric nonlinearity effects can be neglected.

## 3. Results and Discussion

Molecular configurations of alkanethiolate adsorbed on Au(111) were optimized in our DFT calculations. The optimized structural parameters have a good agreement with those in the literature [38]. Here, we further focus on adsorption-induced surface stress and its influence on the deformation and stress field in the micro-cantilever sensor.

### 3.1. Surface Stress from DFT Calculations

The surface stresses from DFT calculations are listed in Table 2. The clean Au(111) surfaces have isotropic tensile stresses of 3.02 N/m. The isotropic and tensile natures of surface stresses for a clean surface, arising from charge accumulation between the surface atoms, have been reported in the literature and our results are in good agreement with these theoretical studies [11].

In contrast, anisotropic surface stresses are found on the alkanethiolate-covered gold surface. For example, the induced surface stresses of hexanethiolate adsorbed on Au(111) are a compressive stress of −1.54 N/m along the molecular chain direction and a tensile stress of 0.28 N/m along a direction perpendicular to the molecular chain. These anisotropic surface stresses can be understood by the following behaviors.

First, the sulfur atom of alkanethiolate attracts electrons from the surface Au atoms to form covalent-like Au–S bonds. Therefore, this charge removal in the gold surface yields a compressive surface stress. The surface stresses of the alkanethiolate with shorter chains are much smaller than those of the clean Au surface, indicating a larger relief of tensile stress or a compressive stress during molecular adsorption. In addition, the formation of two Au–S bonds on the three-hollow site breaks the symmetric structure on the gold surface and yields a huge anisotropic compressive stress.

Second, molecular chains generate attractive forces in this molecular configuration on Au(111). Due to the orientation of the long molecular chain, the attractive force along the direction of the chain is smaller than that in the perpendicular direction. Therefore, larger attractive forces in a direction perpendicular to the molecular chain could yield greater tensile stress and compensate for the compressive stress produced by the charge redistribution. For example, the induced surface stress of −1.54 N/m for hexanethiolate on Au(111) along the direction perpendicular to the molecular chain is much more compressive than the 0.28 N/m stress in the direction parallel to the molecular chain. The average surface stress of hexanethiolate adsorption on the Au(111) surface is a compressive stress of −0.63 N/m and has a very good agreement with experimental data within ±0.05 N/m [39].

### 3.2. Device Deformation and Stress Fields from FEM Analysis

**Figure 6.**
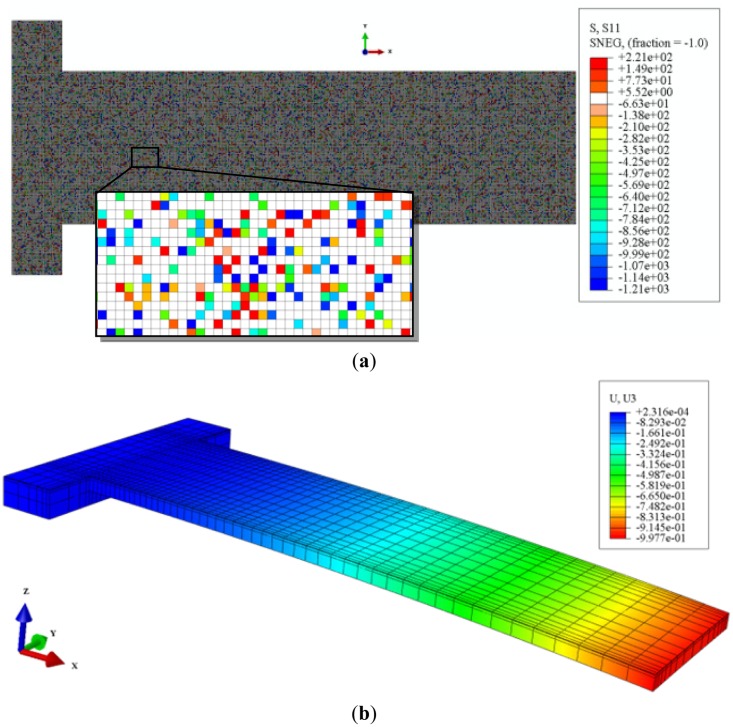
(**a**) Top view of the S_11_ (MPa) stress contour of the shell surface subjected to a 30% coverage of hexanethiolate SAMs on Au(111). The white color indicates no initial stress; (**b**) The Z-component of the displacement (μm) of the micro-cantilever subjected to full coverage of hexanethiolate SAMs on the gold surface.

The principal surface stresses from DFT calculations were uniformly transformed into normal and shear stresses, and then randomly applied to the shell elements in our FEM models as shown in Figure 6a. The range of these stresses was between two principal stresses (Δ*g*_11_ and Δ*g*_22_) divided by the film height, which is the distance between the center of the gold slab and the last carbon in the chain of the optimized alkanethiolate in the DFT unit cell, in a direction normal to the surface. The stress/strain analysis was carried out in ABAQUS. Figure 6b shows an example of the deformation contour of the micro-cantilever sensor.

**Figure 7.**
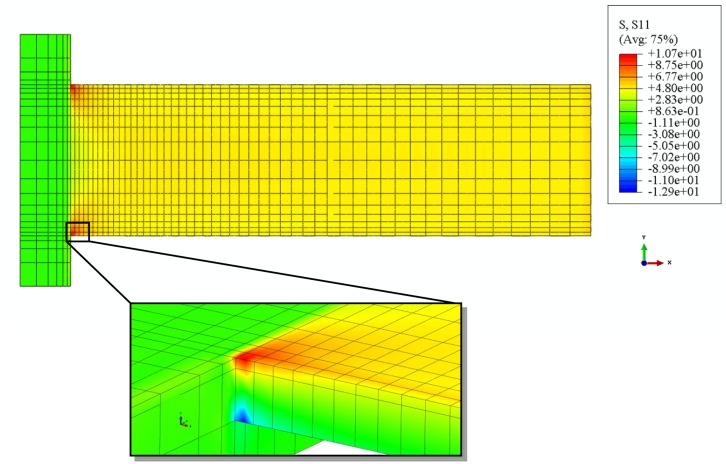
Top view of the S_11_ stress distribution of the silicon nitride layer in the micro-cantilever subjected to a full coverage of hexanethiolate SAMs on Au(111).

The longitudinal stress distribution in the silicon nitride layer of the micro-cantilever subjected to a full coverage of hexanethiolate SAMs on Au(111) is shown in Figure 7. The stress in the supporting block is relatively small compared with those in the beam, where the tensile (compressive) S_11_ stress occurs on the upper (bottom) surface of the silicon nitride layer due to the overall compressive surface stress in the shell section. The S_11_ stresses concentrate near the corners of the cantilever, close to the supporting block, as shown in the inset of Figure 7. The same pattern of stress distribution was found in the micro-cantilever subjected to all the other alkanethiolate SAMs on its top surface. This suggests a good location for embedding piezo-resistive elements. The analytical suggestion is reinforced by the recent experimental work on fabricating a piezo-resistive type microcantilever for detection of DNA from hepatitis B virus [10].

**Figure 8.**
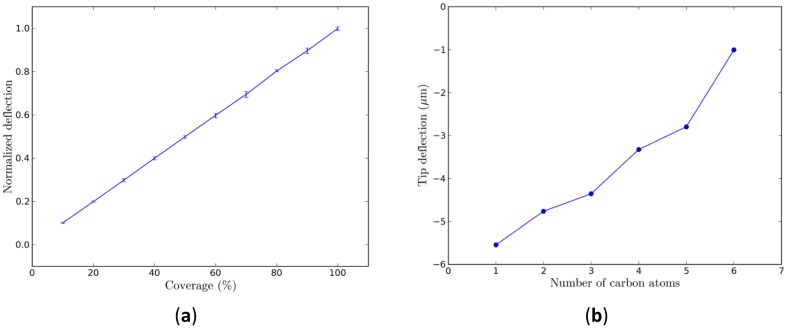
(**a**) Average tip deflection as a function of the coverage of alkanethiolate SAM domains. The error bars indicate the range of the maximum and minimum tip deflections at each sample at the same coverage; (**b**) Average tip deflections as a function of alkanethiolate chain length at full coverage.

Based on a multiscale simulation framework, we can further study the effects of the alkanethiolate SAM coverage or the chain length at the molecular level on the deformation of the micro-cantilever sensor at the device level. For different coverages of SAM domains, an FEM analysis of ten samples of randomly distributed SAM domains was conducted and the average of the tip deflections of these ten samples was used for further analysis. The result is shown in Figure 8a, where we find that the average tip deflection is linearly proportional to the coverage of hexanethiolate SAM domains: the higher the coverage, the greater the deflection. This feature is the same for other alkanethiolates with different chain lengths. The linear coverage dependence with an increase of coverage is expected since the stress contribution of individual adsorbate atoms adds up linearly to result in a macroscopic tip deflection [40]. For fully covered SAM domains on Au(111), however, the average tip deflection decreases as the chain length of the alkanethiolate increases, as shown in Figure 8b. This is expected as the compressive surface stresses generated from longer chain alkanethiolate decrease because of London dispersion forces.

## 4. Conclusions

A multiscale simulation framework for micro-cantilever sensors has been developed. It successfully connected density functional theory (DFT) calculations and finite element method (FEM) analysis to predict the device-level deformation and stress fields. DFT calculations were used to resolve the induced surface stresses of molecular adsorption upon the recognition layer while FEM analysis was conducted to analyze the device response of the nanomechanical sensors due to molecular adsorption. Alkanethiolate self-assembled monolayer (SAM) adsorption upon the Au(111) surface of a micro-cantilever sensor was used as an example to demonstrate the applicability of this multiscale framework. The tip deflection of the micro-cantilever sensors increase linearly as the coverage of SAM domains increases. Under full coverage, the tip deflection decreases as the molecular chain length increases.

The present multiscale simulation framework allows us to predict the overall displacement and stress fields of the device. One immediate benefit by using the present scheme is that molecular calculations of surface stresses such as those from DFT now become relevant for analyzing deformation and stress fields of a microcantilever device. The multiscale framework provides a quantitative analysis of the displacement and stress fields of micro-cantilever sensors, and can be used to predict the responses of nanomechanical sensors subjected to complex molecular adsorption.

## Figures and Tables

**Table 1.** Material properties and thicknesses of each layer of the micro-cantilever in μMKS units.

	**Young's Modulus, *E*(MPa)**	**Poisson's Ratio**	**Thickness (μm)**
Surface Film Layer	60,000	0.4	0.00066–0.00127
Gold	60,000	0.4	0.02
Silicon Nitride	280,000	0.2	0.5
Silicon	160,000	0.3	0.5

**Table 2.** Induced surface stresses of the clean and alkanethiolate-covered Au surfaces. C1, C2, C3, C4, C5, and C6 denote methanethiolate, ethanethiolate, propanethiolate, butanethiolate, pentanethiolate, and hexanethiolate, respectively.

**Surface**	***g*_11_(N/m)**	***g*_22_(N/m)**	**Δ*g*_11_(N/m)**	**Δ*g*_22_(N/m)**
clean	3.02	3.02		
C1-covered	1.72	0.59	−1.30	−2.43
C2-covered	2.01	0.85	−1.01	−2.17
C3-covered	2.28	0.86	−0.74	−2.16
C4-covered	2.61	1.23	−0.41	−1.79
C5-covered	2.92	1.27	−0.10	−1.75
C6-covered	3.30	1.48	0.28	−1.54
